# The novel sustained 3‐hydroxybutyrate donor poly‐D‐3‐hydroxybutyric acid prevents inflammatory bowel disease through upregulation of regulatory T‐cells

**DOI:** 10.1096/fj.202200919R

**Published:** 2022-12-23

**Authors:** Rimina Suzuki, Mayuko Mishima, Masaki Nagane, Hinano Mizugaki, Takehito Suzuki, Mariko Komuro, Takuto Shimizu, Tomoki Fukuyama, Shiro Takeda, Masaya Ogata, Takayoshi Miyamoto, Naoyuki Aihara, Junichi Kamiie, Shinji Kamisuki, Hiroto Yokaryo, Tadashi Yamashita, Takumi Satoh

**Affiliations:** ^1^ School of Veterinary Medicine Azabu University Sagamihara Japan; ^2^ Center for Human and Animal Symbiosis Science Azabu University Sagamihara Japan; ^3^ Okinawa Prefectural Industrial Technology Center Okinawa Japan; ^4^ School of Bioscience and Biotechnology Tokyo University of Technology Hachioji Japan

**Keywords:** 3‐hydroxybutyrate, biodegraded plastic, inflammatory bowel disease, poly‐D‐3‐hydroxybutyric acid, short‐chain fatty acids, Tregs

## Abstract

Inflammatory bowel disease (IBD) is a chronic persistent intestinal disorder, with ulcerative colitis and Crohn's disease being the most common. However, the physio‐pathological development of IBD is still unknown. Therefore, research on the etiology and treatment of IBD has been conducted using a variety of approaches. Short‐chain fatty acids such as 3‐hydroxybutyrate (3‐HB) are known to have various physiological activities. In particular, the production of 3‐HB by the intestinal microflora is associated with the suppression of various inflammatory diseases. In this study, we investigated whether poly‐D‐3‐hydroxybutyric acid (PHB), a polyester of 3‐HB, is degraded by intestinal microbiota and works as a slow‐release agent of 3‐HB. Further, we examined whether PHB suppresses the pathogenesis of IBD models. As long as a PHB diet increased 3‐HB concentrations in the feces and blood, PHB suppressed weight loss and histological inflammation in a dextran sulfate sodium‐induced IBD model. Furthermore, PHB increased the accumulation of regulatory T cells in the rectum without affecting T cells in the spleen. These results indicate that PHB has potential applications in treating diseases related to the intestinal microbiota as a sustained 3‐HB donor. We show for the first time that biodegradable polyester exhibits intestinal bacteria‐mediated bioactivity toward IBD. The use of bioplastics, which are essential materials for sustainable social development, represents a novel approach to diseases related to dysbiosis, including IBD.

**Abbreviations**
3‐HB: 3‐hydroxybutyric acid
AUC: area under the curve
DAI: disease activity index
DSS: dextran sulfate sodium
GPCR: G protein‐coupled receptors
H&E: hematoxylin and eosin
HCA2: hydroxycarboxylic acid receptor 2
HDAC: histone deacetylase
IBD: inflammatory bowel disease
IM: ionomycin
KE: ketone ester
NLRP3: NLR family pyrin domain containing 3
PHB: poly‐D‐3‐hydroxybutyric acid
PMA: phorbol 12‐myristate 13‐acetate
pTregs: periphery‐derived Tregs
SCFA: short‐chain fatty acid
tTreg: thymus‐derived Tregs

## 1 INTRODUCTION

Inflammatory bowel disease (IBD) is a chronic persistent intestinal disorder, with ulcerative colitis and Crohn's disease being the most common.^1^ IBD is involved in the dysfunction of the intestinal barrier, immune system, intestinal microbiota, and genetic factors, but the physiopathology of IBD development is unknown.^1^, ^2^ Therefore, research on the etiology and treatment of IBD has been conducted using a variety of approaches.

Intestinal microbiota are reportedly associated with the development of IBD, and short‐chain fatty acids (SCFAs) produced by butyrate‐producing bacteria can suppress the pathogenesis of IBD.^3^ SCFAs, such as 3‐hydroxybutyric acid (3‐HB) and propionic acid, and are found in feces as metabolites of intestinal bacteria. SCFAs are absorbed into the body by transporters and simple diffusion in the colon and exhibit various physiological activities.^4^ Several molecular mechanisms underlie the physiological activity of 3‐HB, which can act via inhibitory G protein‐coupled receptors (GPCR), such as Hydroxycarboxylic acid receptor 2 (HCA2), FFAR2, and FFAR3, and via inhibitor of histone deacetylase.^3^, ^4^, ^5^, ^6^ SCFAs exhibit proliferative and immunomodulatory effects on intestinal epithelial cells and immune cells, and therefore, can serve as a therapeutic option for IBD.^7^ However, SCFAs are highly volatile and challenging to deliver to the colon via oral administration. An indirect approach using probiotics is currently used to increase SCFAs in the colon as a treatment option for IBD.^2^

For sustainable human development, biodegradable plastics are an attractive material for use. For example, poly‐D‐3‐hydroxybutyric acid (PHB), a polyester of 3‐HB, can be degraded by soil microorganisms.^8^ Although mammalian cells cannot degrade PHB, soil bacteria and the intestinal microflora present in mammalian bodies can degrade PHB to 3‐HB. PHB is a non‐toxic polymer of 3‐HB produced by various bacteria including *Halomonas* sp. and is widely used as a bioplastic in the fields of industry and medical implants.^8^, ^9^ In addition to bioplastic materials, PHB enhances the growth of fish and shrimps by modulating their enterobacteria.^10^, ^11^, ^12^ Recently, several groups have shown that PHB can be used in mammals. Fernandez et al. showed the PHB inhibited carcinogenesis in a rat chemical‐induced colorectal cancer model.^13^ In the micro mini pig, PHB enhances the production of several SCFAs such as acetate, propionate, and butyrate.^9^

Therefore, PHB may serve as a functional food and show various health effects by improving the gut environment in pets, industrial animals, and humans. We hypothesized that PHB has the potential to function as a donor of 3‐HB through degradation by the intestinal microbiota. Because SCFAs such as 3‐HB produced by intestinal bacteria have been reported to exhibit anti‐colitis properties, we aimed to investigate the effect of PHB as a 3‐HB donor on the pathology of IBD.

## 2 MATERIALS AND METHODS

### 2.1 PHB production

PHB was produced using *Halomonas* sp. by a modification of method in previous report.^14^ Briefly, the bacterium was cultivated aerobically at 35 °C in a 90 L fermenter (MSJ‐U2W 90 L, B.E. Marubishi Co., Ltd., Japan) with 40 L medium containing 250 g/L sucrose, 5 g/L K_2_HPO_4_, 5 g/L NaCl, 20 g/L NaNO_3_, 1 g/L K_2_SO_4_, 0.2 g/L MgSO_4_ · 7H_2_O, 0.04 g/L CaCl_2_, 0.01 g/L FeSO_4_ · 7H_2_O, 0.09 g/L EDTA · 4Na, and 1 ml /L trace mineral solution (2.8 g/L H_3_BO_3_, 2.0 g/L MnSO_4_ · 7H_2_O, 0.2 g/L ZnSO_4_ · 7H_2_O, 0.1 g/L CuSO_4_ · 5H_2_O, 0.4 g/L Na_2_MoO_4_ · 2H_2_O, and 0.1 g/L Co(NO_3_)_2_ · 6H_2_O). The agitation speed and aeration rate were maintained at 500 rpm and 80 L per minute, respectively. The cultivated cells were autoclaved and centrifuged at 10 000*g*. PHB powder was obtained after washing the precipitated pellets with water and drying. The number average molecular weight was 201 671 and the average degree of polymerization was 2345, as measured by gel permeation chromatography.

### 2.2 General animal methods

Animal experiments were performed according to the “Law for the Care and Welfare of Animals in Japan” and the ARRIVE guidelines. In addition, all animal experiments were approved by the Animal Experiment Committee of Azabu University (Approval No. 210825‐3). Male C57BL/6 N mice (Charles River Laboratories, Kanagawa, Japan), 8–10 weeks old were used. Mice were housed in plastic cages in an air‐conditioned room at 24°C under a 12 h light–dark cycle (light on at 7:00 a.m.), with food and water available ad libitum under specific pathogen‐free conditions. At the end of the experiment, all animals were euthanized by cervical dislocation using 2% isoflurane anesthesia.

### 2.3 Fecal and serum 3‐HB analysis

Serum 3‐HB was analyzed using a blood 3‐HB detector with a disposable chip (StatStrip, Nova Biomedical, MA, USA) after 2% PHB feeding or oral administration of Ketone ester (KE; (*R*)‐3‐hydroxybutyl (*R*)‐3‐hydroxybutyrate). KE is rapidly hydrolyzed by esterase to 3‐HB and 1,3‐butanediol, which are absorbed through the small intestinal epithelium. Furthermore, 1,3‐butanediol is oxidized by alcohol dehydrogenase in the liver to produce 3‐HB.^15^

Fecal 3‐HB was analyzed via GC–MS following derivatization (oximization and trimethylsilylation). Fecal samples (10 mg) were extracted using 250 μl of methanol/Milli‐Q water/chloroform (5:2:2, v/v/v). Next, 5 μl of the internal standard (2‐isopropylmalic acid, 1 mg/ml) and 40 μl of methoxyamine hydrochloride (40 mg/ml, pyridine solution) were added and allowed to react at 30°C for 90 min. Further, 40 μl of N‐trimethylsilyl‐N‐methyl trifluoroacetamide +1% trimethylsilyl chloride was added and allowed to react at 37°C for 45 min. The reaction mixture (1 μl) was analyzed using a GC–MS system (GC 7890A, Agilent Technologies, Germany) equipped with a 30 m × 0.25 mm DB‐5 ms capillary column with a 0.25 μm film thickness (Agilent Technologies, Germany), an automated sample injector MPS (GERSTEL GmbH & Co. KG, Germany), and MS detector 5975B (Agilent Technologies, Germany).

### 2.4 DSS‐induced colitis model

**FIGURE 1:**
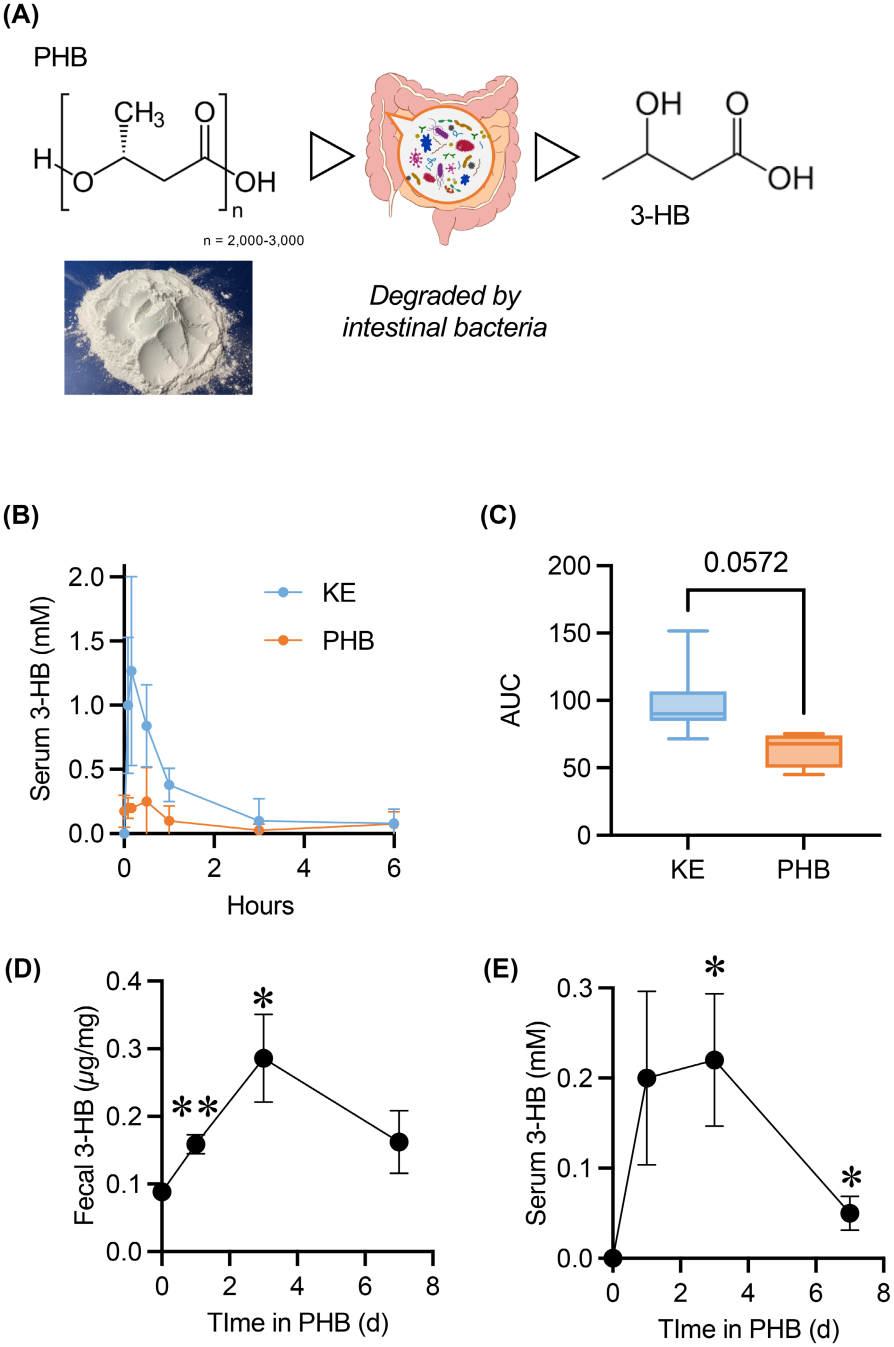
Poly‐D‐3‐hydroxybutyric acid (PHB) caused an increase in serum and fecal 3‐HB levels. (A) Schematic for the degradation of poly‐D‐3‐hydroxybutyric acid by intestinal bacteria. (B) Serum 3‐HB level measured after single oral administration of Ketone ester (KE, 1 g/kg po., equivalent to 2 g/kg of 3‐HB) and PHB (Approx. 2 g/kg/day). (C) Area under the curve analysis for serum 3‐HB after KE and PHB administration. (D) Fecal and (E) blood 3‐HB concentration after administration of 2% PHB containing food (*n* = 8/group, *p < .05, **p < .01).

To induce colitis, mice were administered 2% (w/v) dextran sulfate sodium (DSS, Cat No. 199‐08361, Wako Pure Chemical Industries, Osaka, Japan) in sterile drinking water, which was provided ad libitum as described previously.^16^ The DSS group was fed a normal diet. The DSS + PHB group was treated with DSS and fed a 2% PHB diet as described in Figure 1A. Mice were monitored daily for body weight, diarrhea, and macroscopic bleeding, which were scored using the Disease Activity Index (DAI) as described in a previous study.^17^

### 2.5 Histological and immunohistochemical analysis

The colons of the animals excluding the cecum were prepared as “Swiss rolls”.^18^ The cross‐sections of colon tissue were excised and fixed using 4% neutral buffered formaldehyde, embedded in paraffin, and 4 μm thick sections were prepared, as described previously.^19^ Histopathological analysis was performed using hematoxylin and eosin (H&E) staining. Inflammation was scored as described previously.^20^

For IHC staining, deparaffinized sections of mouse colons were treated with citrate buffer (10 mM sodium citrate, pH 6.0) for heat‐induced antigen retrieval using a pressure cooker (120 Pa, 4°C, 2.5 min). To remove endogenous peroxidase, the slides were immersed in 3% hydrogen peroxide for 10 min and subsequently incubated with blocking buffer (5% goat serum in PBS with 0.1% Triton‐X100) at 4°C for 30 min. For the primary antibody reaction, the slides were probed with rat anti‐FoxP3 monoclonal antibody (catalog no. 14‐5773‐82, Thermo Fisher Science, Waltham, MA, USA, dilution 1:300), rat anti‐CD3 monoclonal antibody (catalog no. ab11089, Cambridge, UK, dilution 1:100), or rabbit anti‐acetylated‐lysine polyclonal antibody (catalog no. #9441, Cell Signaling Technology, Danvers, MA, USA, dilution 1:3000) at 4°C overnight.

For FoxP3 staining, HRP‐conjugated secondary antibodies (catalog no. 414311, Nichirei Bioscience, Tokyo, Japan) were reacted with the sections on the slides for 1 h, and DAB substrate was reacted with the slides for 8 min. Next, tissues were counter‐stained with hematoxylin and mounted. For CD3 and Ac‐K staining, fluorochrome‐conjugated secondary antibodies (dilution 1:1000, catalog no. A48265 and A32731, Thermo Fisher Scientific) were reacted with the slides for 1 h. The tissues were mounted using ProLong Gold antifade reagent with DAPI (catalog no. P36931, Thermo Fisher Scientific). Images were acquired with a BZ‐X700 microscope (Keyence, Osaka, Japan).

### 2.6 Reverse transcription‐quantitative polymerase chain reaction (RT‐qPCR) analysis

RT‐qPCR was performed as previously described with modifications.^21^ Total RNA dissolved from the colon of mice was purified using the Tissue Total RNA Mini Kit (catalog no. FATRK 001; Favorgen Biotech Corporation, Ping‐Tung, Taiwan) according to the manufacturer's instructions. RNA (500 ng) was reverse‐transcribed using the ReverTra Ace qPCR RT Master Mix with gDNA remover (catalog no. FSQ‐301, TOYOBO, Osaka, Japan). Real‐time PCR analysis was performed using a LightCycler®96 system together with a FastStart Essential DNA Green Master (catalog no. 06402712001, Roche Molecular Systems, Inc., Basel, Switzerland). The primer sequences used for qPCR are shown in Table 1. Fluorescence intensity was measured at the end of each extension phase. Relative mRNA expression was derived via normalization to β2‐microglobulin as an internal control using E‐method.^22^

**TABLE 1:** Primers used for reverse transcription–quantitative polymerase chain reaction analysis

Gene	Forward primer (5′‐3′)	Reverse primer (3′‐5′)
Mouse B2m	TTTCTGGTGCTTGTCTCACT	GTTCAGTATGTTCGGCTTCC
Mouse Ccl2	GCCTGCTGTTCACAGTTGC	CTGCTGCTGGTGATCCTCTT
Mouse Foxp3	ACTGGGGTCTTCTCCCTCAA	CAAAAGGTTGCTGTCTTTCC
Mouse Il10	TCCCCTGTGAAAATAAGAGC	TCATTCATGGCCTTGTAGAC
Mouse Nlrp3	TAAGAAGGACCAGCCAGAGT	GAGAGATATCCCAGCAAACC
Mouse Ptgs2	CCAACCTCTCCTACTACACCA	TCCTTATTTCCCTTCACACC
Mouse Tnf	CCCCAAAGGGATGAGAAGTT	CACTTGGTGGTTTGCTACGA

### 2.7 Flow cytometry

Spleens were collected from mice, mechanically disrupted, and isolated splenocytes were stained as described previously.^16^ The antibodies used for flow cytometry are listed in Table 2. For Treg(s) staining, the Treg Detection Kit (catalog no. 130‐120‐674, Miltenyi Biotech, Bergisch Gladbach, Germany) was used according to the manufacturer's instructions. Fluorescence intensities were analyzed using a Cell Analyzer EC800 (Sony Biotechnology, Tokyo, Japan) and FlowJo software (BD Biosciences, Franklin Lakes, NJ, USA).

**TABLE 2:** Antibodies used for flow cytometry

Antibody	Vender: Catalog No.	Dilution
CD3e‐FITC	Thermo Fisher Scientific: 11‐0031‐85	1:100
CD4‐PerCP/Cy5.5	Biolegend: 100434	1:100
CD8‐APC	Thermo Fisher Scientific: 17‐0081‐82	1:200
CD44‐PE	TONBO biosciences: 50‐0441	1:200
CD62L‐PE/Cy7	Biolegend: 104417	1:200
FoxP3‐PE	Biolegend: 12‐5773‐80	1:50
CD25‐APC	Biolegend: 102011	1:50

### 2.8 Cell culture and luciferase assays

Jurkat cells (Cat No. RCB3052, Riken Cell Bank, RIKEN Cell Bank, Tsukuba, Japan) and FoxP3 Leeporter Luciferase Reporter‐Jurkat cells (catalog no. 14‐144ACL, Abeomics, CA, USA) were cultured in RPMI1640 medium (catalog no. 189‐02025, Wako Pure Chemical) supplemented with 10% FBS and 10 mM HEPES. Luciferase assays were performed using the Leeporter Luciferase Assay Reagent (catalog no. #17‐1101, Abeomics) according to the manufacturer's instructions. Luminescence was quantified using the GloMax‐Multi Detection System (Promega, Fitchburg, WI, USA). Cells were stimulated by PMA (catalog no. P1585, Sigma‐Aldrich, St. Louis, MO) and Ionomycin (IM, catalog no. 095‐05831, Wako Pure Chemical) along with KE treatment.

### 2.9 Western blot

Western blot was performed as described in a previous study.^21^ Cells were lysed using modified RIPA Buffer and subjected to sonication with BIORUPTOR® II TYPE6 (Cat. No. BR2006A, BM Equipment Co., Ltd., Tokyo, Japan). We loaded 10–20 μg of protein into the wells of the SDS‐PAGE and transferred them to a PVDF membrane (Cat. No. IPVH 00010, Merck Millipore, Burlington, MA) for 1 h at 100 V using Towbin buffer (25 mM Tris, 192 mM glycine, 20% MeOH). The membrane was blocked at 4°C for 1 h using 5% skim milk containing TBST and probed with primary antibodies (Table 3) at 4°C overnight. The secondary antibody, horseradish peroxidase (HRP)‐conjugated goat anti‐rabbit or mouse IgG (1:20 000; Cat. No. A16110/A16078, Thermo Fisher Scientific), was used at 4°C for 1 h. For the loading control, HRP‐conjugated anti‐GAPDH (1:20 000; HRP‐60004, Proteintech) was used. Images were acquired using Immobilon Western chemiluminescent HRP substrate (Cat. No. WBKLS0500, Merck Millipore) and an iBright imager (Thermo Fisher Scientific).

**TABLE 3:** Antibodies used for Western blot

Antibody	Vender: Catalog No.	Dilution
Acetylated‐Lysine	Cell Signaling Technology: #9411	1:10 000 in 5% BSA/TBST
AcK9/14‐Histone H3	Cell Signaling Technology: #9677	1:50 000 in 5% BSA/TBST
Histone H3	Proteintech: 17168‐1‐AP	1:50 000 in 5% BSA/TBST
AcK5/8/12/16‐Histone H4	Abcam: ab177790	1:50 000 in 5% BSA/TBST
Histone H4	Genetex: GTX129560	1:5000 in 5% BSA/TBST
FoxP3	Thermo Fisher Scientific: 14‐7979‐82	1:1000 in 5% BSA/TBST
TUBB	Proteintech: HRP‐66240	1:50 000 in 5% skim milk/TBST

### 2.10 Statistical analysis

All values are expressed as box‐and‐whisker plots from 4–8 independent experiments. The Student's *t*‐test (two‐sided) or Tukey multiple *t*‐test were used to analyze differences between groups, and significance was evaluated at *p* < .05. Data were analyzed using Prism 9 (GraphPad Software, San Diego, CA, USA).

## 3 RESULTS

### 3.1 PHB is a sustained donor of ketone bodies

PHB can be degraded by the microflora in the intestinal tract in a manner similar to that which occurs in the soil (Figure 1A). After a single oral administration of KE, a donor of 3‐HB, a rapid increase in blood 3‐HB levels was observed at 5–10 min after administration of KE (1 g/kg, equivalent to 2 g/kg of 3‐HB) and 2% PHB containing food (~2 g/kg/day) with slightly increased 3‐HB levels (Figure 1B). In addition, the AUC of PHB was reduced compared to that of KE upto 12 h (Figure 1C). We next measured 3‐HB concentrations in feces and blood at 1–7 days after PHB feeding. A significant increase in 3‐HB concentrations was observed in the fecal and serum samples of the PHB feeding group at 1–7 days (Figure 1D,E), and no significant increase in 3‐HB concentrations (feces and serum) was observed after 4 or 8 weeks (data not shown). These results indicate that oral administration of PHB results in its degradation in the intestinal tract, where it acts as a donor to release 3‐HB in the feces and serum.

### 3.2 PHB suppresses the pathology of the DSS‐induced IBD model

**FIGURE 2:**
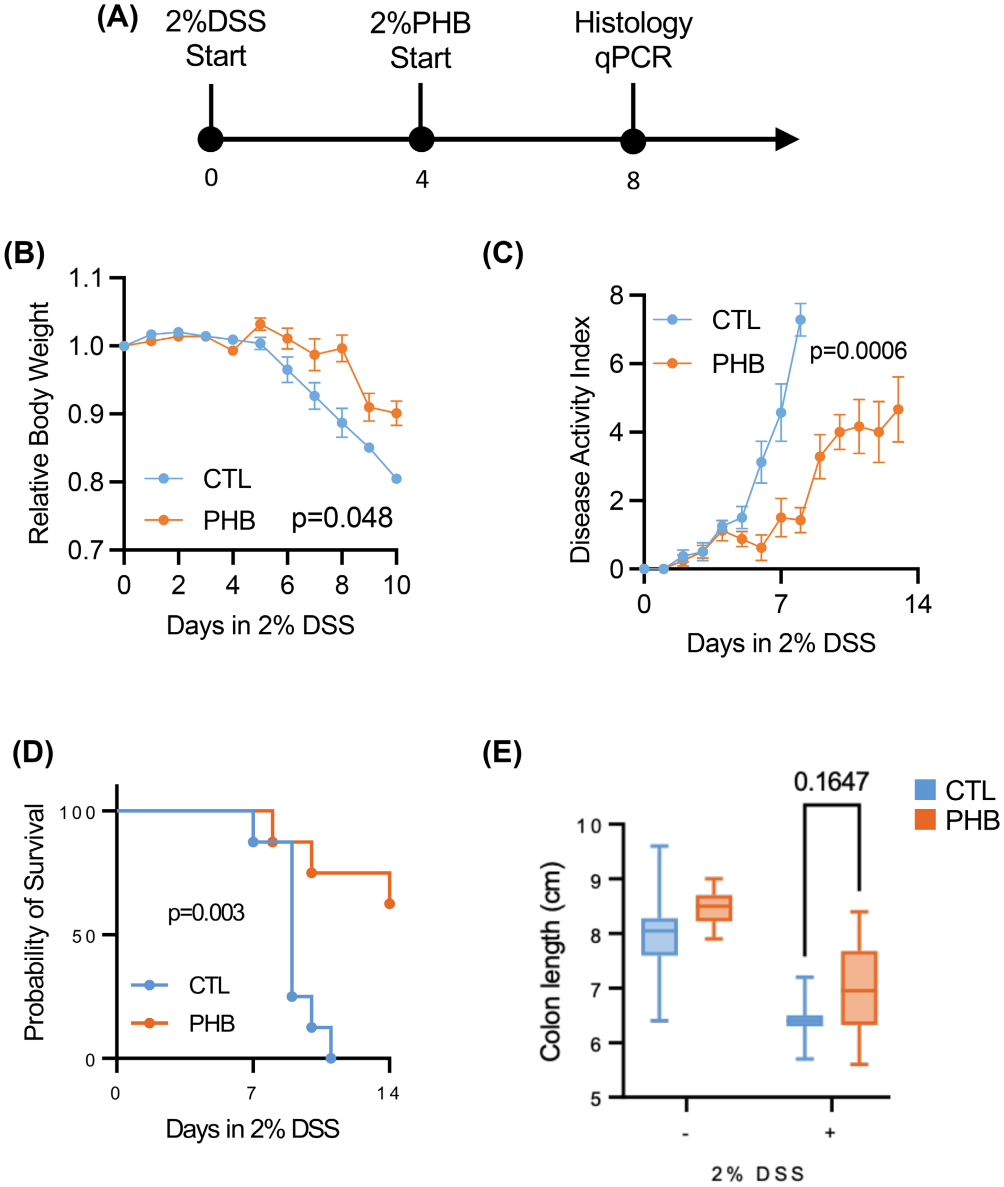
Poly‐D‐3‐hydroxybutyric acid (PHB) prevented the pathology of the dextran sulfate sodium (DSS)‐induced IBD model. (A) Mice were orally administered 2% DSS via drinking water to induce colitis. At four days after DSS treatment, 2% poly‐D‐3‐hydroxybutyric acid (PHB) was administered and the (B) body weight, (C) DAI scores were observed daily and (D) survival rate. (E) Colon length was analyzed at 8 days after 2% DSS treatment (*n* = 8/group; *p < .05).

SCFAs produced by intestinal bacteria can alleviate the pathophysiology of IBD.^3^ To examine the effects of a PHB diet on the prevention of IBD, the PHB diet was fed ad libitum from day 4 of DSS treatment (Figure 2A). In the control group, weight loss, increased DAI scores, and bloody stools were observed after DSS administration; however, in the PHB group, weight loss and decreased DAI scores were observed after DSS was administered ad libitum (Figure 2B,C). In addition, the survival rate was prolonged in the PHB group (Figure 2D). On the other hand, the PHB group did not suppress colon shortening associated with DSS administration (Figure 2E).

**FIGURE 3:**
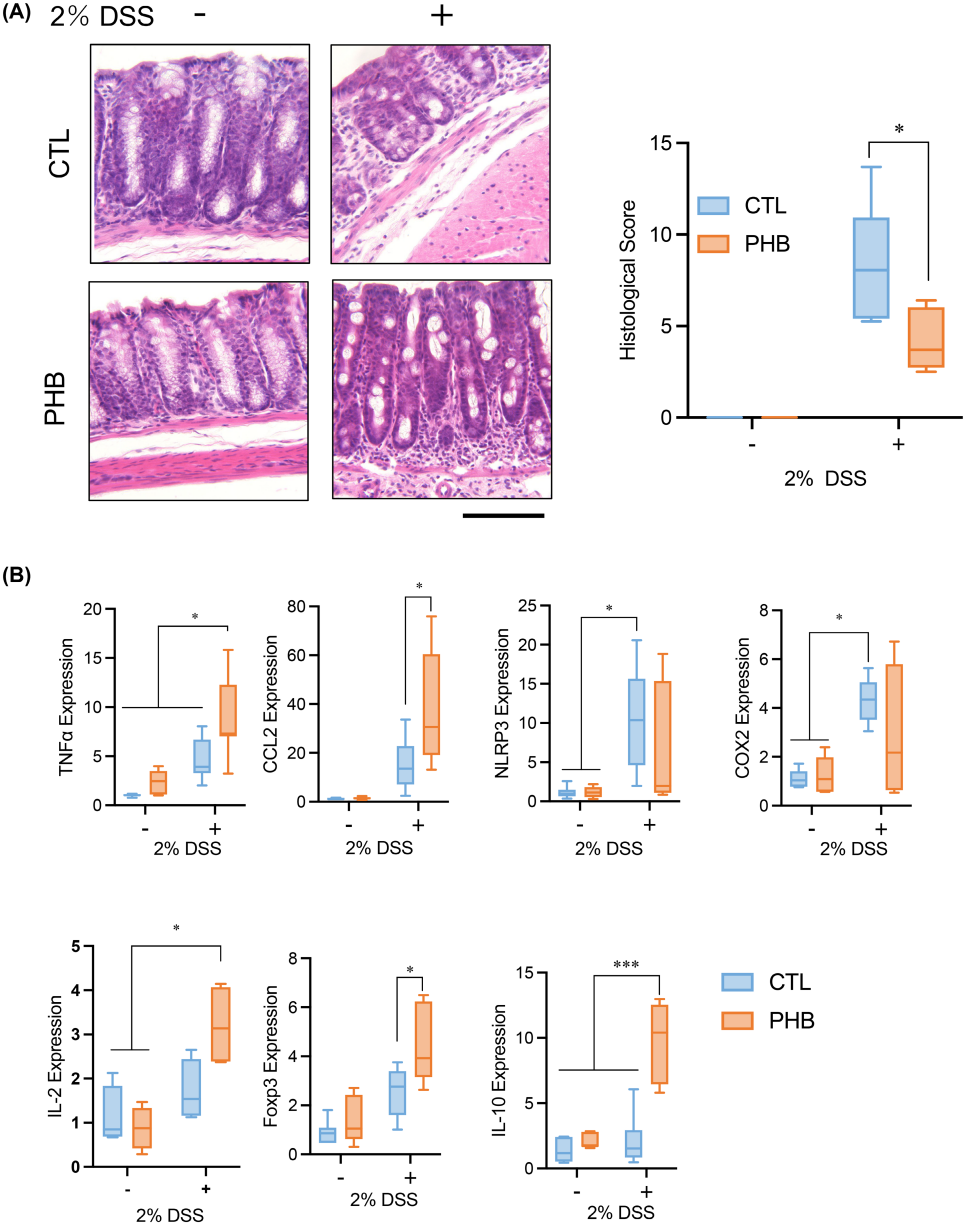
Poly‐D‐3‐hydroxybutyric acid (PHB) suppressed histological inflammation in the dextran sulfate sodium (DSS)‐induced IBD model. (A) Histopathology was examined by hematoxylin and eosin staining of colon sections of mice eight days after 2% DSS treatment, and the histological scores were analyzed by evaluating 20 fields per slide. Scale bar = 100 μm. (B) Gene‐expression levels of TNFα, CCL2, Cox2, NLRP3, FoxP3, IL‐2, and IL‐10 in the colon tissue were analyzed eight days after 2% DSS treatment (*n* = 8/group; *p < .05, **p < .01, ***p < .001).

Analysis of histological inflammation after eight days of DSS administration showed that the PHB group had suppressed inflammation scores (Figure 3A). Gene expression of the inflammatory cytokines (TNFα and CCL2) was slightly increased in the DSS and DSS + PHB groups. We observed a significant increase in inflammation markers (NLRP3 and COX2) in the DSS group but not in the DDS + PHB group. In addition, gene expressions of the anti‐inflammatory cytokine (IL‐10 and IL‐2) and Foxp3 were increased in the DSS + PHB group (Figure 3B). IL‐10 is important in regulating the development of intestinal inflammation.^23^ These results indicated that PHB treatment suppresses histological inflammation via IL‐10 production.

### 3.3 PHB induces periphery derived Tregs

**FIGURE 4:**
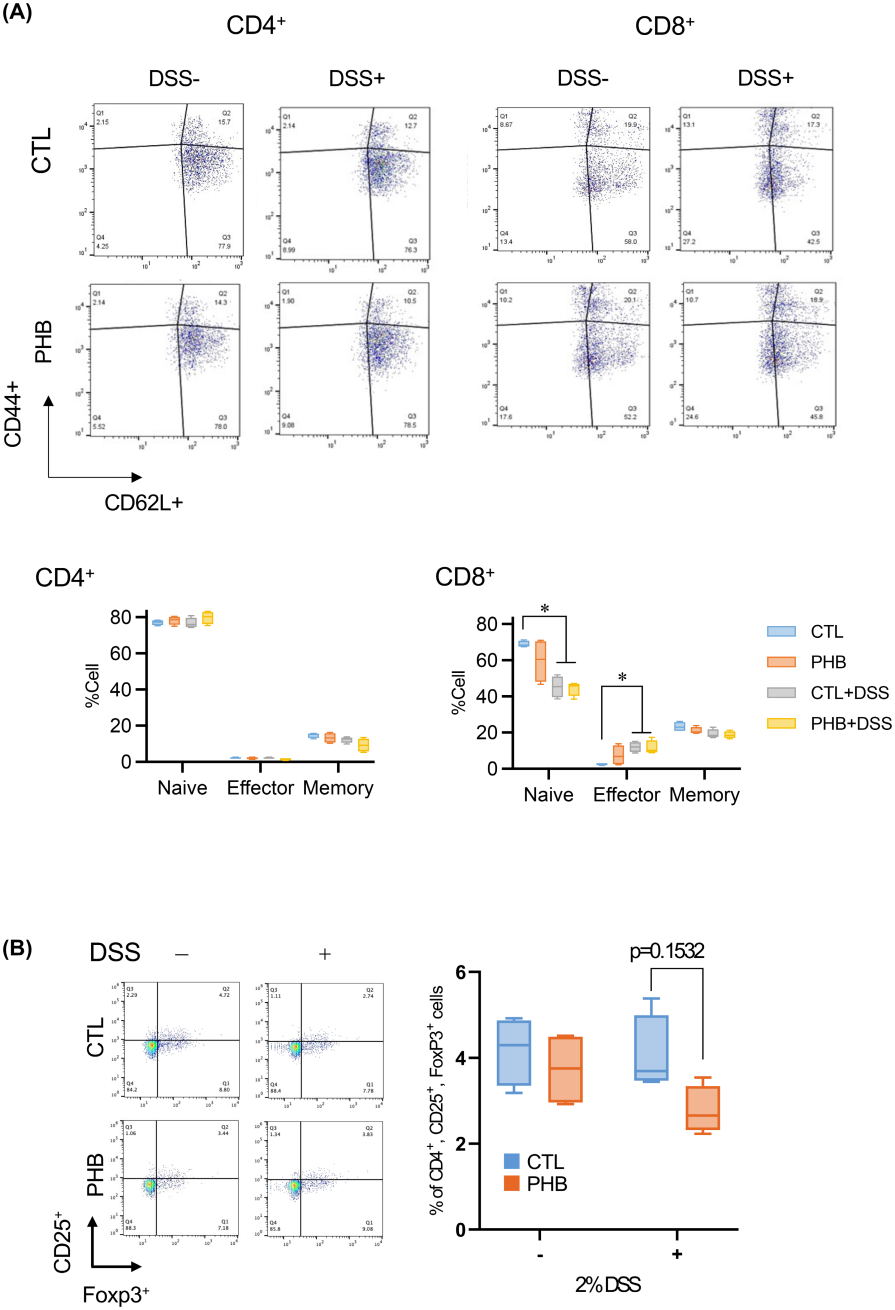
Poly‐D‐3‐hydroxybutyric acid (PHB) was not involved in the effector function of T cells in the spleen. Splenic CD4^+^ or CD8^+^ T cells were isolated from mice eight days after 2% DSS treatment. (A) Representative flow cytometry plots and cumulative data from individual mice depicting the percentages of naive, effector, and memory cells. (B) Representative flow cytometry plots and cumulative data from individual mice depicting the percentages of CD4^+^CD25^+^FoxP3^+^ cells (*n* = 4/group; *p < .05).

T cells play an important role in the pathogenesis of IBD and are the target of various therapeutic agents. 3‐HB affects T cell function^24^; therefore, we investigated the effect of PHB on T cells. Analysis of T cell subsets in the spleen showed no variation in the percentage of memory or effector T cells after DSS and PHB were administered ad libitum (Figure 4A). Therefore, no significant variation in the profile of T cell subsets in the systemic circulation was observed during this study period. IL‐10 is mainly released from regulatory T cells and Foxp3 gene expression were increased in the DSS + PHB group. We analyzed Tregs (CD4^+^, CD25^+^, FoxP3^+^) in the spleen after treatment with DSS. The proportion of Tregs in the spleen decreased in the DSS group and in the DSS + PHB group (Figure 4B).

**FIGURE 5:**
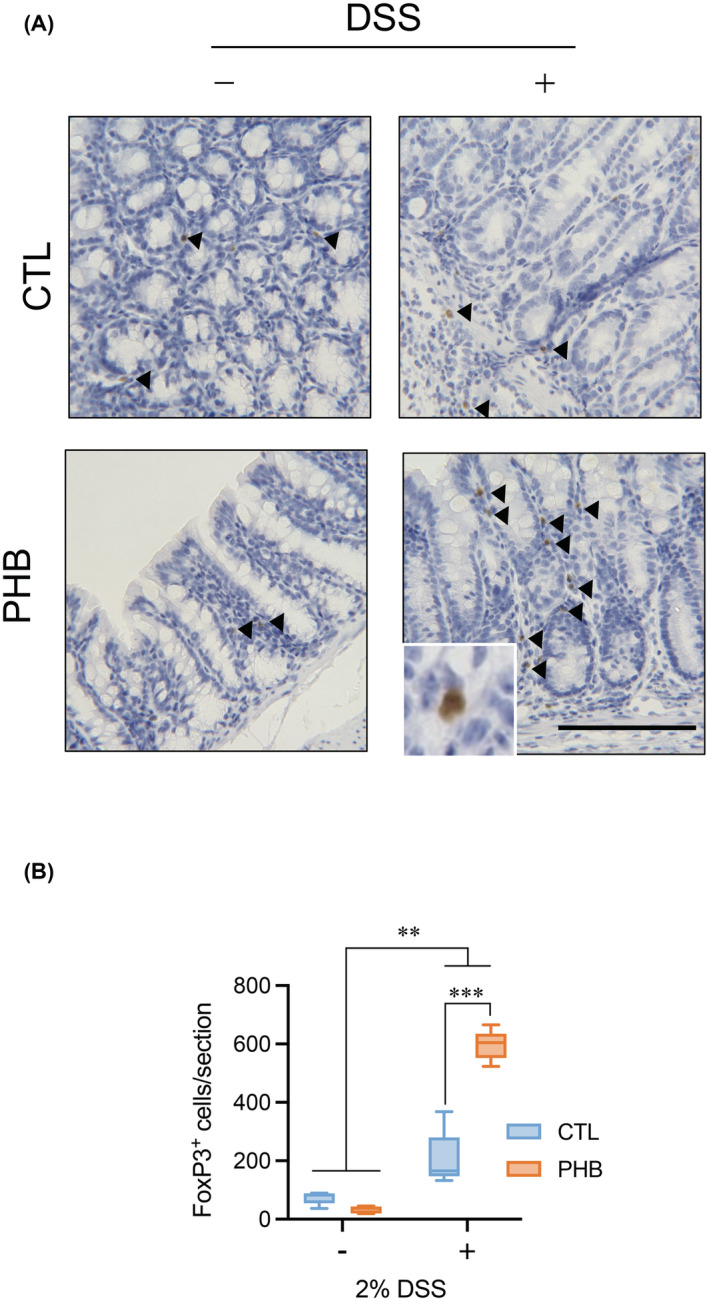
Poly‐D‐3‐hydroxybutyric acid (PHB) increased peripheral Tregs in the dextran sulfate sodium (DSS)‐induced IBD model. (A) Representative images of FoxP3 immunohistochemical staining of colon sections eight days after 2% DSS treatment. Scale bar = 100 μm. (B) FoxP3 positive cells (black arrowheads) per section were counted (*n* = 5/group; **p < .01, ***p < .001).

Tregs originate in the thymus and by differentiation in the periphery.^24^, ^25^ The distribution of Tregs in the colon tissue was subsequently analyzed by immunohistochemistry, and the number of Tregs in the rectum was increased in the DSS‐treatment group. PHB treatment also caused a significant increase in the number of Tregs in the rectum compared to DSS group (Figure 5A).

### 3.4 PHB and KE treatment enhances FoxP3 activity

**FIGURE 6:**
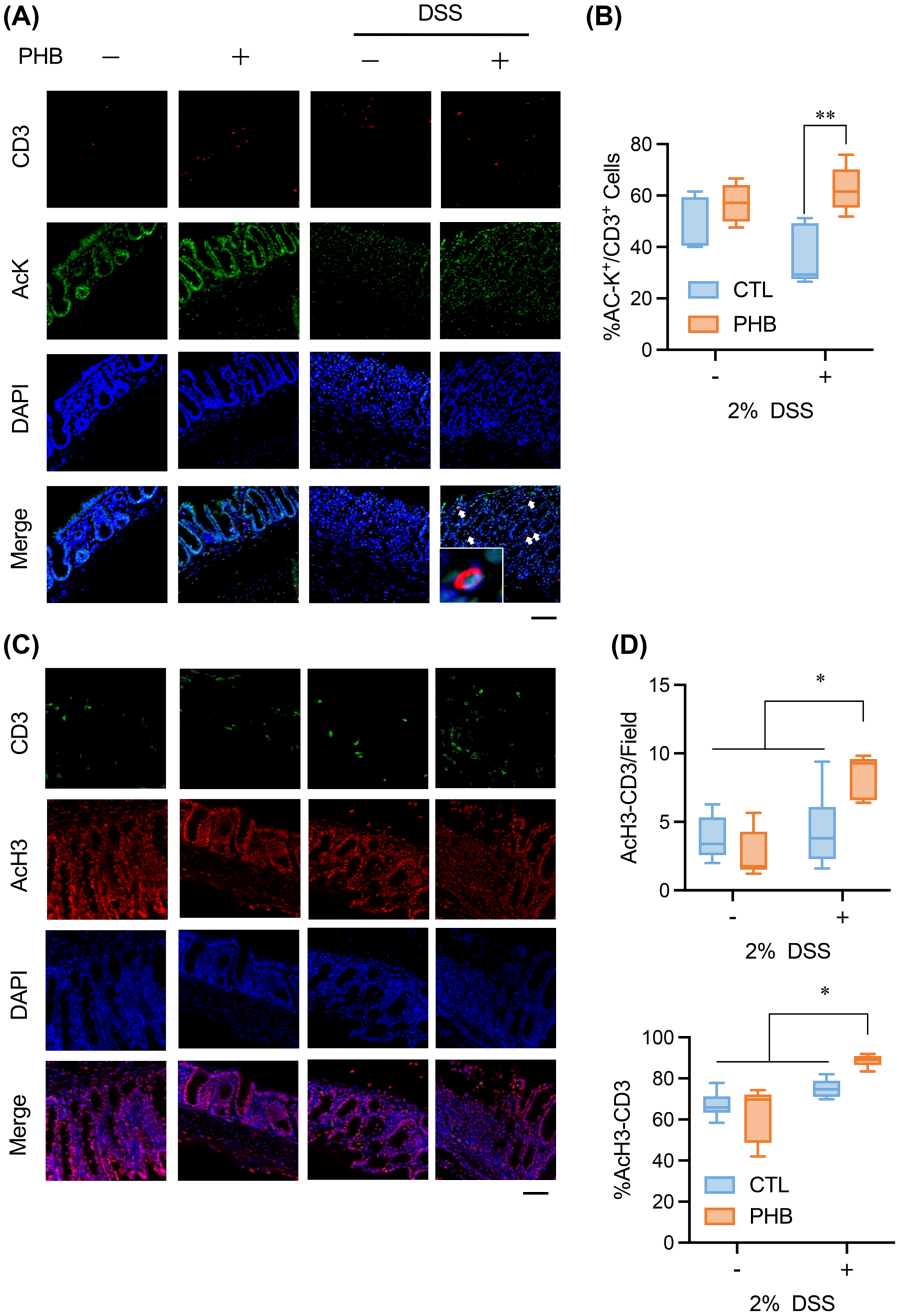
Poly‐D‐3‐hydroxybutyric acid (PHB) increased tissue acetylation levels. (A) Representative images of immunohistochemical staining for CD3 (red) and Ac‐K (green) in colon sections eight days after DSS treatment. White arrow indicates CD3 Ac‐K double positive cells. Scale bar = 100 μm. (B) Ratio of Ac‐K positive CD3^+^ cells of the rectum per section were counted (*n* = 5/group; *p < .05). (C) Representative images of immunohistochemical staining for CD3 (green) and Ac‐Histone H3 (Ac‐H3, green) in colon sections eight days after DSS treatment. (D) Ac‐Histone H3 positive CD3^+^ cells of the rectum per field were counted, and calculated the percentage of Ac‐H3 positive CD3^+^ cells in CD3^+^ cells (*n* = 5/group; *p < .05).

3‐HB has HDAC inhibitory activity and increases protein acetylation levels. Therefore, we analyzed the cell types that are acetylated by PHB in the intestine after DSS treatment. As shown in Figure 6A, CD3‐positive cells in the rectum were acetylated after PHB treatment (Figure 6A,B). Analysis of acetylated Histone H3 in T cells was performed because of the prominent acetylated lysine detected in their nuclei. The number of acetylated Histone H3‐positive T cells tended to increase in tissues in the DSS group, and significantly increased in the PHB + DSS group. The percentage of acetylated Histone H3‐positive T cells in the tissues also increased (Figure 6C,D).

**FIGURE 7:**
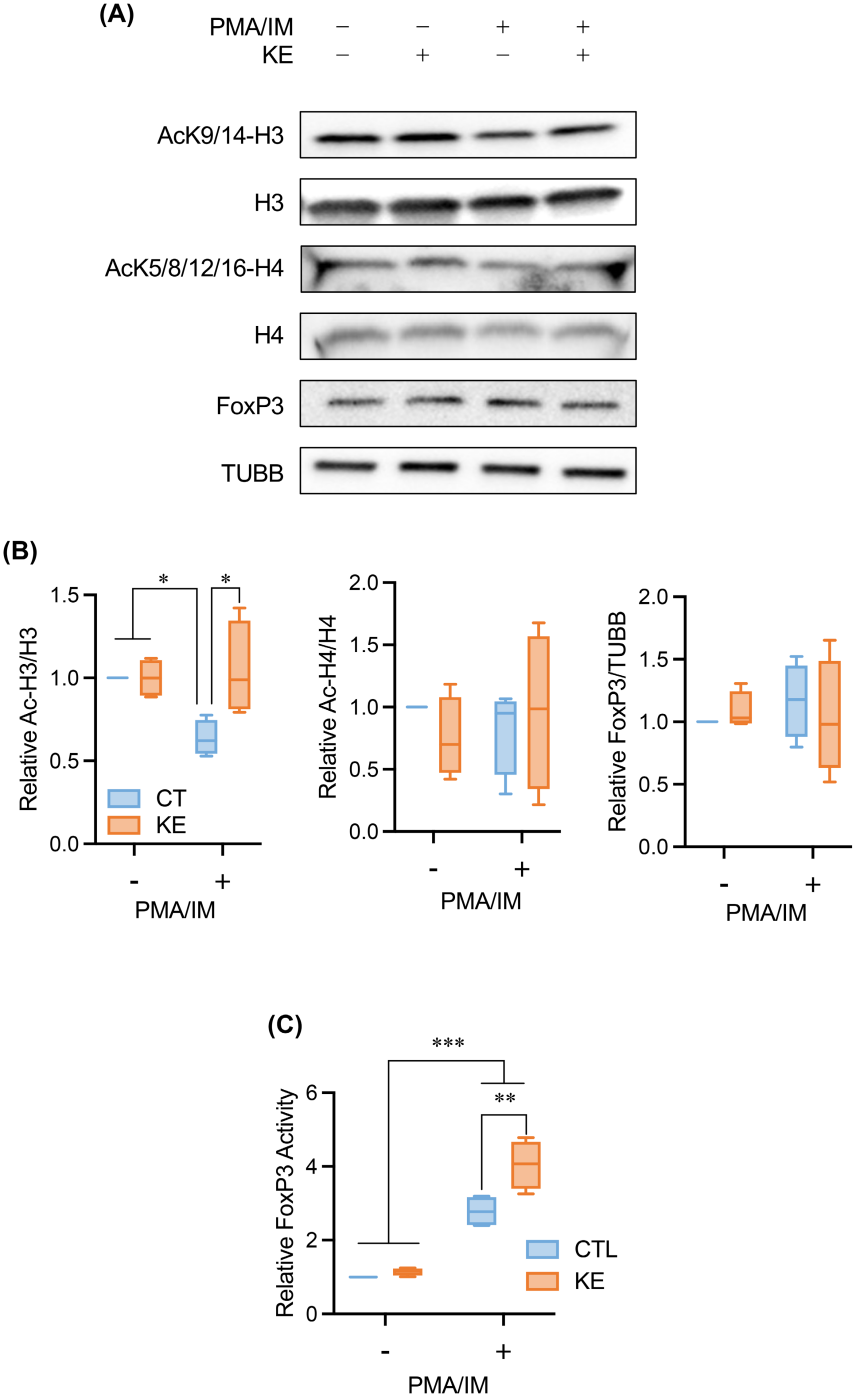
Ketone ester increased acetylated Histone H3 level and FoxP3 activity. Jurkat cells were treated for 24 h with Ketone ester (KE, 500 μM), followed by stimulation (0.2 nM PMA and 0.4 μM IM) for 24 h with KE. (A) Representative images and (B) quantitative data of acetylated Histone H3, H4, FoxP3 after stimulation with KE. (C) Transcriptional activity of FoxP3 was evaluated using reporter assay (*p < .05, **p < .01, ***p < .001).

Stimulation with PMA/IM decreased the acetylation level of Histone H3 in the Jurkat cells. In comparison, pretreatment with KE increased the acetylation level of Histone H3 after PMA/IM (Figure 7A,B). We did not observe the effects of PMA/IM or KE treatment on Histone H4 acetylation and FoxP3 expression. Moreover, KE treatment‐enhanced transcriptional activity of FoxP3 was observed after PMA/IM stimulation by reporter assay (Figure 7C). These results suggest that 3‐HB released from PHB increases Histone H3 acetylation and FoxP3 activity, leading to the accumulation of Tregs.

## 4 DISCUSSION

Ketone bodies, represented by 3‐HB, are alternative energy sources that are mobilized when the body is depleted of glucose, which is an essential energy source in the body.^15^, ^26^, ^27^ Ketones are produced through strict dietary restrictions, and their therapeutic effects have been reported for various diseases such as epilepsy,^28^ diabetes,^29^ and cancer.^30^ However, due to dietary restrictions, KD reportedly has multiple side effects,^31^, ^32^ posing high barriers to its clinical application.

KE, a commonly used 3‐HB donor, is rapidly degraded in the small intestine.^15^, ^33^, ^34^ As shown in Figure 1, KE induces a sharp increase in 3‐HB within several minutes, suggesting that it is an effective donor, but the increase is not sustained. In contrast, PHB induced a sustained and significant increase within several days. As PHB is expected to function in the lower gastrointestinal tract because it is degraded by bacteria in the large intestine, we propose that PHB may act as an effective and sustained 3‐HB donor system, which may be useful for the treatment of chronic diseases.

PHB was developed as a biodegradable plastic and is used as a material for food containers and tableware. Its use is increasing due to its low environmental impact. In this study, we have shown for the first time that biodegradable resins are degraded by intestinal microflora, and that the resulting monomers constituting the polyesters exhibit biological effects. However, blood 3‐HB levels decreased to the level found before administration of PHB despite 1–2 weeks of PHB feeding (data not shown). This result could be attributed to fluctuations in the microflora that metabolize PHB. Further studies are required to clarify the effect of PHB on the microbiome in long‐term treatment cases.

Further, we demonstrated that PHB has an anti‐IBD effect (Figure 3). PHB feeding inhibited weight loss and increased clinical scores in a DSS‐induced IBD mouse model. Multiple results for SCFAs on Tregs have been reported, including 3‐HB produced by the gut microbiota, which promotes Treg accumulation. Tregs produce anti‐inflammatory cytokines such as IL‐10 and exhibit anti‐IBD effects by suppressing excessive immune responses.^35^ As shown in Figure 3, PHB feeding increased Tregs and IL‐10 gene expression in the colon tissue. Although FoxP3‐positive cells include thymus‐derived Tregs (tTreg) and periphery‐derived Tregs (pTregs),^25^ PHB feeding did not suppress the decrease in Tregs in the spleen, which is associated with IBD pathology, indicating an accumulation of pTregs. Because regulatory T cells are deeply involved in the pathogenesis of IBD, the increase in Tregs by PHB may serve as the basis for their physiological activity.

**FIGURE 8:**
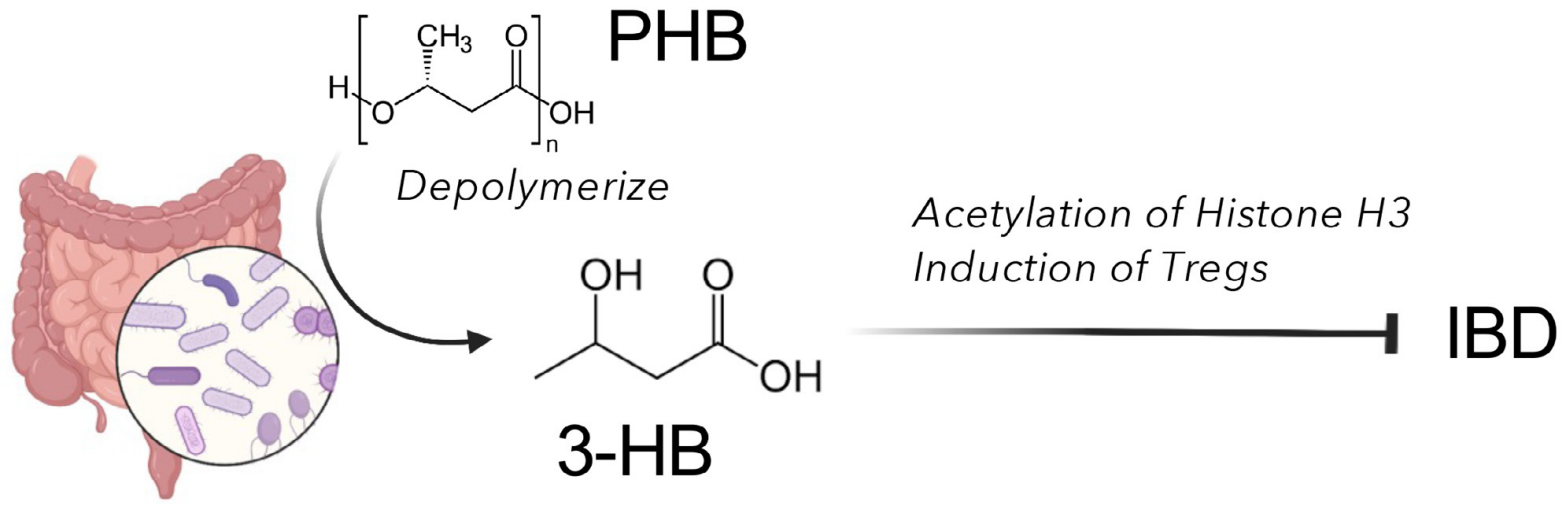
Proposed mechanism of PHB against IBD. PHB is hydrolyzed in the large intestine by depolymerase released from microbiota. Released 3‐HB acts on both mammals and microbiota in the lumen of the large intestine. Here, we found that HDAC inhibition may be mainly involved in the activation of Tregs in the gut.

In addition to its GPCR‐mediated action, 3‐HB inhibits histone deacetylases. The acetylation status of histones regulates FoxP3 activity.^36^ Several reports indicate that 3‐HB is an endogenous HDAC inhibitor implicated in intestinal epithelial integrity.^37^ In addition, HDAC inhibitors SAHA and trichostatin A have been reported to have Treg‐mediated anti‐IBD effects,^37^, ^38^, ^39^ suggesting that 3‐HB produced by PHB degradation functions as an HDAC inhibitor in the intestinal tract. In this study, PHB feeding induced the acetylation of CD3‐positive cells and increased the number of Tregs in the colon (Figure 6). To examine the specific target protein of 3‐HB, we focused on the acetylation status of Histone H3. As shown in Figure 6C,D, PHB increased in acetylated Histone H3‐positive T cells. Moreover, KE enhanced the Histone H3 acetylation and transcriptional activity of FoxP3 in Jurkat cells after PMA/IM stimulation (Figure 7). These results suggest that 3‐HB produced from PHB/KE acts in the periphery to promote Treg accumulation (Figure 8).

Along with FoxP3 activation by HDAC inhibitory effect, 3‐HB reportedly binds to HCA2 within physiological concentrations and induces potent anti‐inflammatory actions in the colon. Thus, activation of HCA2 might be involved in the inhibition of IBD by PHB. In addition to the direct 3‐HB donor action described above, the effect of PHB on the gut microbiota must be considered. In micropigs, PHB has been reported to increase SCFAs other than 3‐HB in feces, such as propionic acid, butyric acid, and acetic acid.^9^ PHB can produce only 3‐HB but no other SCFAs, suggesting that the other SCFAs are produced by intestinal bacteria activated by PHB. Therefore, 3‐HB released from PHB may affect SCFA‐producing bacteria in the gut and enhance their activity. These SCFAs may activate Treg under the epithelium of the large intestine.^24^, ^36^ Based on this, a detailed investigation of the involvement of PHB in the intestinal microbiota will be necessary in the future.

Currently, there are three available 3‐HB donors: KE, 3‐hydroxybutyrate sodium salt (3‐HBNa), and PHB. KE and 3‐HBNa have high bioavailability and their use is highly limited in the large intestine because they are absorbed in the small intestine. In contrast, PHB is not digested in the small intestine and hydrolyzed in the large intestine. In future studies, the functionality of PHB, a highly novel 3‐HB donor, should be investigated in various disease models. In conclusion, we show for the first time that biodegradable plastics exhibit intestinal bacteria‐mediated bioactivity toward IBD. The use of bioplastics, which are essential materials for sustainable social development, represents a novel approach to the treatment of diseases related to the intestinal microbiota, including IBD and other diseases.

## AUTHOR CONTRIBUTIONS

All authors contributed to the study's conception and design. Rimina Suzuki and Masaki Nagane performed material preparation, data collection, and analysis. Rimina Suzuki and Masaki Nagane wrote the first draft of the manuscript. Mayuko Mishima and Masaki Nagane wrote the revised manuscript. All authors have read and approved the final manuscript. Conceptualization: Masaki Nagane and Takumi Satoh. Methodology: Rimina Suzuki, Mayuko Mishima, Hinano Mizugaki, Mariko Komuro, Takuto Shimizu, Takehito Suzuki, Masaya Ogata, Tomoki Fukuyama, Naoyuki Aihara, Junichi Kamiie, and Hiroto Yokaryo. Formal analysis and investigation: Rimina Suzuki, Masaki Nagane, Takehito Suzuki and Takumi Satoh. Resources: Takumi Satoh, Shinji Kamisuki, and Hiroto Yokaryo. Writing—original draft separation: Rimina Suzuki and Takumi Satoh; Writing—review and editing: Rimina Suzuki and Masaki Nagane. Funding acquisition: Masaki Nagane.

## FUNDING INFORMATION

This work was supported by the Japan Society for the Promotion of Science (JSPS) KAKENHI Grant Numbers 17K08140, 19K20452, and by the Center for Human and Animal Symbiosis Science (Azabu University).

## DISCLOSURES

Masaki Nagane, Tadashi Yamashita, and Takumi Satoh are applying for the patent for the use of PHB.

## Data Availability

The data that support the findings of this study are available in the methods and/or supplementary material of this article.
